# Acetate Signalling Regulates Virulence-Associated Traits in the Esca Pathogen *Phaeomoniella chlamydospora*

**DOI:** 10.3390/jof12070539

**Published:** 2026-07-22

**Authors:** Ádám Novák, Dóra Szabó, Adrienn Gomba-Tóth, Nikolett Molnár, Kálmán Zoltán Váczy, Zoltán Karácsony

**Affiliations:** Food and Wine Research Institute, Eszterházy Károly Catholic University, Leányka s. 8/G, H-3300 Eger, Hungary; novak.adam@uni-eszterhazy.hu (Á.N.); szabo.dora@uni-eszterhazy.hu (D.S.); molnar.nikolett@uni-eszterhazy.hu (N.M.); vaczy.kalman@uni-eszterhazy.hu (K.Z.V.); karacsony.zoltan@uni-eszterhazy.hu (Z.K.)

**Keywords:** *Phaeomoniella chlamydospora*, grapevine trunk diseases, acetate, quorum-sensing, pathogenesis

## Abstract

*Phaeomoniella chlamydospora* (Pch) is a pioneer pathogen of esca, one of the most destructive grapevine trunk diseases worldwide. A recent work suggests that acetate may act as a quorum-sensing (QS) molecule in Pch, promoting biofilm formation in a concentration-dependent manner. However, the broader influence of acetate on virulence-associated traits remains unexplored. In this study, three Pch isolates were cultured under increasing sodium acetate concentrations (0–100 mM) and assessed for pigmentation, extracellular enzyme activities (amylase, cellulase, protease, esterase, and pectinase), phenolic compound-degrading capacity, and antibacterial activity against a grapevine-associated *Pseudomonas* sp. isolate. Pigmentation, as well as amylase and cellulase activities, were significantly increased at low acetate supplement levels (6.25–12.5 mM), while esterase activity was unaffected. The expression of these traits decreased above 25 mM acetate supplementation. Phenolic compound degradation capacity, antibacterial efficacy, as well as protease and pectinase activities progressively suppressed at all acetate concentrations. These results indicate that acetate concentration modulates multiple virulence-associated phenotypes of Pch in vitro. Based on these patterns, we propose a hypothetical model in which acetate-dependent phenotypic changes may reflect a shift between establishment-associated activities and reduced extracellular activity at higher acetate levels. This model remains to be validated by mechanistic and *in planta* infection-based studies.

## 1. Introduction

Grapevine trunk diseases (GTDs) represent one of the most devastating threats to viticulture worldwide, causing estimated annual losses exceeding $1.5 billion globally due to vine decline, reduced productivity, and premature vineyard replanting [1,2]. Among these diseases, esca stands out as particularly destructive and complex, having plagued European vineyards for centuries yet remaining incompletely understood [3,4]. At the centre of the esca disease complex lies *Phaeomoniella chlamydospora* (Pch), a slow-growing, darkly pigmented ascomycete that colonizes the xylem tissues of grapevines and is considered a pioneer pathogen initiating the disease cascade. The pathogen colonizes the xylem vessels of grapevines, where its growth leads to dark brown wood streaking and vascular occlusion [5]. Esca is now understood as a disease complex involving the synergistic action of multiple fungal pathogens at different stages of disease progression [6]. In young vines, Pch and species of *Phaeoacremonium*, particularly *P. minimum* (formerly *P. aleophilum*) [7], cause Petri disease, a vascular disorder characterized by brown-black wood discoloration, reduced vigour, and decline [8]. In mature vines, these tracheomycotic fungi are joined by the basidiomycete *Fomitiporia mediterranea* [9], which causes a white rot of the wood, completing the classical esca triad [3,10]. Foliar symptoms of esca include the distinctive interveinal chlorosis and necrosis known as “tiger-stripe” leaf patterns, termed apoplexy, which manifests as the sudden and complete wilting of entire vines during hot summer periods [2,3]. The economic impact of esca is staggering: in France alone, approximately 12% of vineyards are rendered economically unviable, with annual losses estimated at €1 billion; in California, losses exceed US $260 million per year [2]. The ban of sodium arsenite—the only partially effective chemical treatment—in 2003 further exacerbated the problem [11].

Despite decades of research, the mechanisms by which Pch establishes infection and contributes to symptom expression remain incompletely understood. A significant advance in this regard was the recent demonstration that Pch is capable of forming biofilms [12]. Biofilm formation—the ability of microorganisms to attach to surfaces and develop structured communities encased in a self-produced extracellular matrix (ECM)—has long been recognized as a critical virulence mechanism in pathogenic fungi, particularly in *Candida albicans* and *Aspergillus fumigatus* [13,14]. In *C. albicans*, biofilms consist of adhesion-competent yeast cells, filamentous hyphae, and an ECM composed of β-1,3-glucan, mannan, proteins, and extracellular DNA, which together confer enhanced resistance to antifungal agents and host immune defences [15,16]. The study by Karácsony et al. [12] revealed that Pch exhibits dimorphic growth in vitro, with yeast-like cells at colony centres embedded in a polysaccharide-rich ECM, transitioning to filamentous growth at colony margins. Critically, this biofilm-forming capacity was also observed on grapevine host tissues used as sole nutrient sources, suggesting that biofilm production occurs *in planta* and may contribute to the occlusion of xylem vessels and consequent disruption of sap flow that characterizes esca pathogenesis [17]. Earlier ultrastructural and fluorescence microscopy studies had documented the colonization of xylem vessels by Pch, including its association with tyloses and gel deposits within the vascular lumen [5,18], patterns consistent with biofilm-like colonization. Among wood-associated fungi, biofilm formation has also been documented in the wood-staining ascomycete *Ophiostoma piceae*, which forms mixed fungal-bacterial biofilms modulated by quorum sensing (QS) signals [19]. The QS refers to a cell density-dependent communication mechanism whereby microorganisms produce, secrete, and detect small signalling molecules—termed autoinducers—that coordinate collective behaviours once a population threshold is reached [20]. First described in bacteria, QS was subsequently identified in fungi with the landmark discovery of farnesol as a QS molecule in *C. albicans* that inhibits the yeast-to-hypha morphological transition [21]. A complementary positive signal, tyrosol, was later shown to stimulate germ tube formation and promote filamentous growth in *C. albicans* [22]; biofilm-associated cells produce significantly higher levels of tyrosol than planktonic cells [23]. Beyond *Candida*, QS phenomena have been described in diverse fungi, including *Histoplasma capsulatum*, *Ceratocystis ulmi*, *Neurospora crassa*, and several filamentous species, involving molecules such as butyrolactones, oxylipins, and aromatic alcohols [24,25]. Cross-kingdom QS interactions further add complexity: the *Pseudomonas aeruginosa* QS molecule 3-oxo-C12-homoserine lactone suppresses *C. albicans* filamentation at biofilm-relevant concentrations, illustrating how bacterial signals can directly modulate fungal morphogenesis [26]. In wood-colonizing environments, where fungi and bacteria coexist in close proximity, such inter-kingdom chemical signalling is likely to shape community dynamics and pathogenic outcomes [19]. Remarkably, a recent study [12] suggests that acetate possibly functions as a QS molecule in Pch that promotes yeast-like growth and ECM production in a concentration-dependent manner. This finding represents the first identification of a putative QS molecule in a grapevine trunk disease pathogen and possibly establishes a link between fungal chemical communication, biofilm formation, and disease biology.

Beyond biofilm formation, successful colonization and symptom development by Pch are likely to involve a broader repertoire of virulence-associated traits, including the production of melanin pigments, secretion of extracellular hydrolytic enzymes, degradation of host-derived phenolic defence compounds, and suppression of competing microorganisms. Although several of these traits have been well characterized as virulence determinants in other fungal pathogens, their specific roles in Pch pathogenesis remain largely unexplored. Melanin pigmentation is a well-established virulence factor in numerous fungal pathogens. Melanins—dark, high-molecular-weight pigments synthesized via the 1,8-dihydroxynaphthalene (DHN) or L-3,4-dihydroxyphenylalanine (L-DOPA) pathways—protect fungal cells against reactive oxygen species (ROS), ultraviolet radiation, enzymatic degradation, and host immune defences [27]. In phytopathogenic fungi such as *Magnaporthe oryzae* and *Colletotrichum* sp., DHN-melanin is essential for generating the turgor pressure required for appressorial penetration of host tissues, and melanin-deficient mutants show dramatically reduced virulence [27,28]. Pch synthesizes the phytotoxic secondary metabolites scytalone and isosclerone as intermediates of the DHN-melanin biosynthetic pathway [29], and the characteristically dark pigmentation of Pch hyphae and chlamydospores is consistent with melanin deposition in the cell wall. However, the functional contribution of melanin to Pch virulence—whether through protection against host-generated ROS during xylem colonization, facilitation of cell wall integrity under osmotic stress, or enhancement of persistence in woody tissues—has not been experimentally addressed. The secretion of hydrolytic enzymes—including cellulases, pectinases, proteases, esterases, and amylases—constitutes a major virulence strategy in plant pathogenic fungi, enabling the degradation of host cell wall barriers, nutrient acquisition from plant tissues, and facilitation of tissue colonization, as demonstrated in, e.g., *Fusarium graminearum* [30]. Comparative genomic analyses of grapevine trunk pathogens revealed that Pch possesses gene families encoding carbohydrate-active enzymes and other secreted hydrolases, although the extent of gene family expansion in Pch was notably smaller than that observed in more aggressive trunk pathogens such as *Neofusicoccum parvum* or *Eutypa lata* [31]. Consistent with these genomic data, earlier histopathological studies reported no visible degradation of lignified cell walls in wood colonized by Pch, in contrast to the pronounced soft-rot patterns caused by *E. lata* [31]. Nonetheless, transcriptomic analysis of Pch co-cultured with *V. vinifera* callus cells revealed significant upregulation of genes encoding plant cell wall-degrading enzymes, including two cellulose-degrading glycoside hydrolases, a pectinesterase, and an extracellular lipase, indicating that these enzymatic functions are actively induced during host interaction even when nutrient supply is not limiting [32]. Furthermore, a biocontrol-related transcriptomic study showed that putative Pch virulence genes related to carbohydrate-active enzymes and secondary metabolite biosynthesis were significantly expressed *in planta*, reinforcing the notion that these enzymatic functions participate in host colonization [33]. A study by Fleurat-Lessard et al. [34] suggested that Pch secretes lignin-modifying enzymes—including lignin peroxidase, manganese peroxidase, and laccase—in infected grapevine wood. However, their conclusions are questionable, since the immunological detection of the lignolytic enzymes was carried out with polyclonal antibodies raised against proteins of a distant basidiomycetous fungus. Despite the lack of clear evidence of lignin-degrading enzymes in Pch, the capacity of the fungus to degrade or detoxify host-derived phenolic compounds represents a potentially important virulence attribute. Grapevine tissues accumulate a range of phenolic defence compounds in response to fungal infection, including stilbenes, flavonoids, and hydroxycinnamic acids, which function as antimicrobial phytoalexins and structural reinforcements of cell walls [35,36]. Fischer et al. [32] identified the upregulation of a dienelactone hydrolase gene in Pch during interaction with grapevine cells—an enzyme class involved in the biodegradation of toxic aromatic compounds—suggesting that Pch possesses enzymatic mechanisms for detoxifying plant phenolics. In addition, Osti et al. [37] proposed that Pch generates hydroxyl radicals mediated by secreted low-molecular-weight metabolites through a Fenton-like reaction. Hydroxyl radicals are extremely reactive, and Pch is likely to apply them to degrade lignin and soluble host phenolics in addition to the previously examined crystalline cellulose [37]. The potential antibacterial activity of Pch metabolites warrants consideration in the context of the polymicrobial xylem environment. Grapevine trunks harbor complex communities of bacteria and fungi, and competitive interactions among these microorganisms are likely to influence colonization dynamics and disease outcomes [19]. While the antibacterial properties of Pch have not been systematically investigated, the diverse array of polyketide compounds produced by this pathogen [29] raises the possibility that some metabolites may function as antimicrobial agents that confer a competitive advantage within the xylem niche.

This study aimed to investigate the effects of acetate on the expression of various putative virulence-related traits in Pch to reinforce the role of the previously hypothesized acetate-mediated QS in the pathogenesis of esca. The present work seeks to advance our understanding of the molecular basis underlying esca disease and to identify potential targets for novel disease management strategies.

## 2. Materials and Methods

### 2.1. Strains and Growth Conditions

The Pch strains were maintained on Potato Dextrose Agar (PDA; Merck KGaA, Darmstadt, Germany), while the *Pseudomonas* sp. strain was maintained on Luria–Bertani (LB) medium. The isolates were preserved in 50% *v*/*v* glycerol (VWR Chemicals, LLC, Solon, OH, USA) at −80 °C. The Pch and *Pseudomonas* sp. strains used in this study are presented in Table 1.

To test the effects of acetate on Pch strains, media amended with 0, 6.25, 12.5, 25, 50, and 100 mM sodium acetate were used. An acetate salt was applied instead of pure acetic acid to prevent significant effects on media pH. Sodium acetate slightly increased the pH from 7.11 (0 mM supplement) to a maximum of 7.21 (100 mM supplement) as measured in PDA medium, while our previous study showed that the physiology of Pch is significantly affected in this acetate concentration range [12]. In addition, the applied concentrations contain both higher and lower values compared to the previously measured ~13 mM maximum natural acetate production of Pch [12]. 

### 2.2. Isolation and Identification of Microbial Strains

Bacterial isolates were obtained from grapevine trunks (*Vitis vinifera* cv. Cabernet Sauvignon) located in the vineyard of Eszterházy Károly Catholic University, Eger, Hungary. Wood chips were collected using a disinfected fine drill bit. The samples were transferred into 100 mL of LB (Luria–Bertani) broth and incubated at 25 °C under 120 RPM continuous agitation for 7 days. Following incubation, the suspension was spread onto LB agar plates to obtain single colonies, which were subcultured to obtain pure isolates. Total genomic DNA extraction was performed according to the following protocol: 200 µL of bacterial liquid culture prepared in 2 mL LB broth was pelleted by centrifugation (10,000 RPM, 5 min, Velocity 15µ microcentrifuge, Dynamica Scientific Ltd., Livingston, UK), the supernatant was removed, and the pellet was frozen at −80 °C for 20 min. Upon thawing, the pellet was washed once with 1 mL of distilled water, vortexed, and centrifuged again (10,000 RPM, 5 min). The pellet was resuspended in 500 µL of lysis buffer (0.5× TAE electrophoresis buffer, 5% *v*/*v* glycerol, Bio-Rad Laboratories GmbH, Munich, Germany) and incubated at 85 °C for 45 min. Following centrifugation (10,000 RPM, 5 min), the supernatant containing genomic DNA was transferred to a new Eppendorf tube (Eppendorf SE, Hamburg, Germany) and stored at −20 °C. The 16S rRNA regions were amplified using 16S rRNA-f (5′-GGTCTGAGAGGATGATCAGT-3′) and 16S rRNA-r (5′-TTAGCTCCACCTCGCGGC-3′) primers synthesized by Thermo Fisher Scientific (Waltham, MA, USA). Reaction products were checked by agarose gel electrophoresis and documented using a BioDocAnalyze gel documentation system (Biometra GmbH, Göttingen, Germany). Amplicons were sequenced (BaseClear B.V., Leiden, The Netherlands), and the sequences were compared in online databases using BLAST (www.ncbi.nlm.nih.gov accessed on 18 June 2024). Bacterial strains were identified based on sequence similarity. Antibacterial assays were performed on methylene blue-containing plates as described previously [39] to identify isolates susceptible to Pch P46 strain metabolites. Strains of Pch were isolated and identified as described previously [12].

### 2.3. Pigmentation Test

To investigate Pch pigmentation, strains were inoculated on potato dextrose agar (PDA, 2% *m*/*v* glucose, 0.4% *m*/*v* potato infusion, 1.5% *m*/*v* agar) medium. Acetate (Fluka, Sigma-Aldrich Chemie GmbH, Buchs, Switzerland) was added to aliquots of the medium to achieve final concentrations of 0, 6.25, 12.5, 25, 50, and 100 mM. The surface of the media was inoculated with 25 µL droplets of Pch conidial suspensions at a concentration of 5 × 10^5^ conidia/mL collected from PDA cultures. Incubation was carried out at 25 °C for 2 days in the dark, followed by 12 days under ambient light conditions in the lab to promote melanin production. All inoculations were done in duplicate. To quantify melanisation, the plates were photographed using an EOS 1100D digital camera (Canon Inc., Tokyo, Japan), and image analysis was performed. Whole colony areas were analysed, and average pixel intensities were used as raw data. Differences from the possible maximum intensity (255) were calculated, resulting in values directly referring to darker coloration.

### 2.4. Assay of Extracellular Enzymatic Activities

YS2 basic medium [1% *m/v* yeast extract (Scharlab S.L., Sentmenat, Barcelona, Spain), 2% *m/v* sucrose (VWR Chemicals, LLC, Solon, OH, USA), and 2% *m/v* bacteriological agar (VWR Chemicals, LLC, Solon, OH, USA)] was used to assay the various enzymatic activities of Pch strains. The medium was supplemented with specific substrates for activity detection: 0.1% *m*/*v* starch for amylase, 0.1% *m*/*v* carboxymethyl cellulose (CMC, Sigma-Aldrich Chemie GmbH, Steinheim, Germany) for cellulase, 10% *v*/*v* commercially available skim milk for protease, and 0.1% *m*/*v* polygalacturonic acid (Sigma-Aldrich Chemie GmbH, Steinheim, Germany) for pectinase activities. For the assessment of esterase activity, the YS2 medium was supplemented with 0.1% *v*/*v* tributyrin (Fluka Chemie AG, Buchs, Switzerland) and emulsified with Tween-80 (Apollo Scientific Ltd., Stockport, UK; 0.1 volume relative to tributyrin) and distilled water (10 volumes relative to tributyrin) prior to adding to the media. Additionally, acetate was added to the media to achieve final concentrations of 0, 6.25, 12.5, 25, 50, and 100 mM. The plates were spot inoculated with 7 µL of Pch conidial suspensions as detailed in Section 2.3. A density of 1 × 10^6^ conidia/mL was used, except for the esterase assay, where a concentration of 5 × 10^6^ conidia/mL was applied to ensure distinct clearance zones. All inoculations were done in triplicate. Following incubation at 25 °C for 7 days, protease and esterase activities were detected by the formation of visible clearance zones. For amylase, cellulase, and pectinase activities, the plates were flooded with Gram’s iodine solution (0.33% *m*/*v* iodine, 0.67% *m*/*v* potassium iodide) to detect clearance zones around the colonies after 10 min of incubation. The plates were photodocumented, and the images were analysed by measuring the radius of enzyme activity zones in mm.

### 2.5. Assessment of Phenolic Compound Degrading Ability Using Fluorescein

The phenolic compound-degrading ability of Pch was assessed in 2.5 mL liquid YS5 medium (1% *m*/*v* yeast extract, 5% *m*/*v* sucrose) using 24-well plates (VWR International, Radnor, PA, USA). The medium was supplemented with acetate to achieve final concentrations of 0, 6.25, 12.5, 25, 50, and 100 mM. Pch strains were inoculated at a concentration of 1 × 10^5^ conidia/mL and incubated for 7 days at 25 °C. Following the 7-day incubation, fluorescein solution (VWR International Kft., Debrecen, Hungary) was added to the samples to a final concentration of 5 µM, and the cultures were incubated for an additional 24 h at 25 °C. Fluorescein was used as a target molecule since Pch does not show activity towards widely used phenolic colour-substrates like tannic acid or ABTS [40], while fluorescein is susceptible to Fenton-degradation [41], a virulence process proposed in Pch [37]. All inoculations were done in triplicate. Subsequently, to improve quantification accuracy, 200 µL aliquots were transferred from the 24-well plates to 96-well plates (VWR International, Radnor, PA, USA) in technical duplicates. The samples were photo documented under UV illumination using a BioDocAnalyze gel documentation system (Biometra GmbH, Göttingen, Germany) equipped with a Canon EOS 1100D digital camera (Canon Inc., Tokyo, Japan), and fluorescence intensity was measured by image analysis. Percent decrease in fluorescence intensities of the inoculated samples relative to an uninoculated control was calculated to express fluorescein degradation.

### 2.6. Determining Antibacterial Efficacy

The antibacterial activity of Pch isolates was assessed in liquid YG medium (1% *m*/*v* yeast extract, 2% *m*/*v* glucose) supplemented with acetate to achieve final concentrations of 0, 6.25, 12.5, 25, 50, and 100 mM. Pch strains were inoculated at a density of 5 × 10^6^ conidia/mL and incubated for 7 days at 25 °C under static conditions. Fermentation broths were filter-sterilized using 0.45 µm syringe filters (Corning Life Sciences, Tewksbury, MA, USA) and mixed in a 1:1 ratio with *Pseudomonas* sp. cultures that had been grown in double-strength Luria–Bertani (LB) broth for 2 days at 25 °C with 120 RPM shaking. Medium controls (containing only sterile broth) and bacterial controls (containing only bacterial culture and YG medium in 1:1 ratio) were also included. After a subsequent 2-day incubation at 25 °C, the optical density was measured at 600 nm using a JASCO V-650 UV–Vis spectrophotometer (JASCO Corporation, Tokyo, Japan).

### 2.7. Software

Statistical comparisons were performed with GraphPad Prism 8.0.2. software (GraphPad Software, San Diego, CA, USA, www.graphpad.com accessed on 1 May 2024) using one-way ANOVA with Tukey’s post hoc test. For image analysis, Fiji software v1.54f was used [42].

## 3. Results

### 3.1. Effect of Acetate on Pigment Formation in Pch

**Figure 1:**
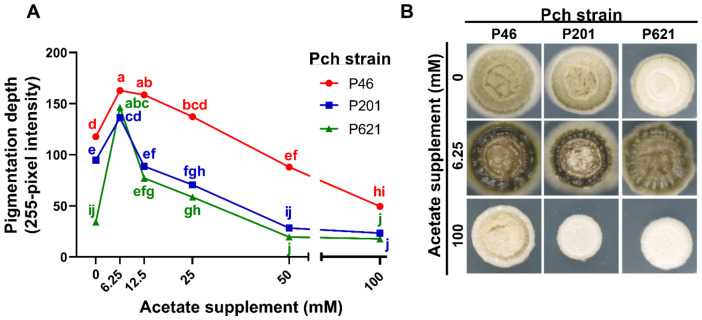
Pigmentation of P46, P201 and P621 Pch strain colonies as a function of acetate supplement concentration in the 0–100 mM range. (**A**) Pigmentation depth of Pch colonies plotted against acetate supplement concentration. Letters mark significantly (*p* < 0.05) differing datasets according to ANOVA. Different colours correspond to the different fungal strains. (**B**) Representative photographs of Pch colonies grown on PDA at 25 °C for 14 days.

The Pch colonies exhibited the typical green-brownish pigmentation characteristic of this species on PDA (Figure 1B). Distinct changes in pigmentation were observed depending on the concentration of acetate supplemented to the media (Figure 1). At low to moderate concentrations (6.25–12.5 mM), pigmentation appeared more intense than in the control (0 mM acetate). At higher concentrations (≥25 mM), pigmentation decreased progressively, with colonies showing paler coloration and reduced radial growth (Figure 1B). Image-based quantification of colony pigmentation, expressed as mean pixel intensity, confirmed the visual trends (Figure 1A). Higher mean pixel values (corresponding to darker pigmentation) were recorded at 6.25 and 12.5 mM acetate. In contrast, colonies exposed to 25–100 mM acetate showed significantly lower pixel values, reflecting inhibited pigment accumulation.

### 3.2. Influence of Acetate on the Exoenzyme Activity of Pch

**Figure 2:**
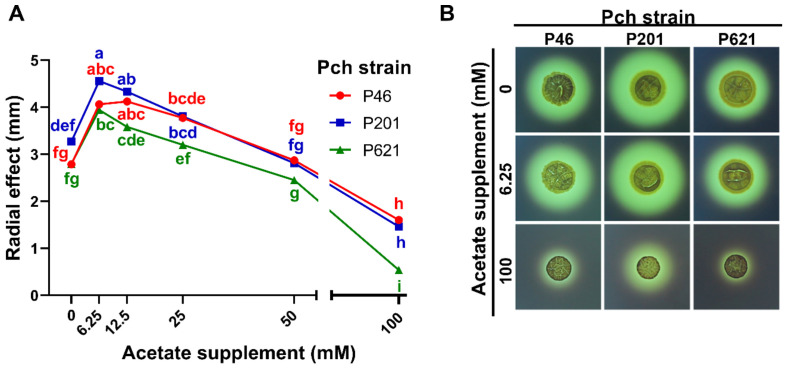
Amylase enzymatic activity of P46, P201 and P621 Pch strain colonies as a function of acetate supplement concentration in the 0–100 mM range. (**A**) Amylase activity radius of Pch colonies plotted against acetate supplement concentration. Letters mark significantly (*p* < 0.05) differing datasets according to ANOVA. Different colours correspond to the different fungal strains. (**B**) Representative photographs of Pch colonies grown on YS2 amended with 0.1% *m*/*v* starch at 25 °C for 7 days. Colonies were stained with Gram’s iodine.

Five extracellular enzymes—amylase, cellulase, protease, esterase, and pectinase—were assayed. Amylase activities can be clearly detected around the Pch colonies (Figure 2B). At low acetate concentrations (6.25–12.5 mM), the activity of all Pch strains significantly increased compared to colonies grown without acetate supplementation (Figure 2A). Amylase activities fell close to the base level in the presence of 25 mM acetate, while 100 mM acetate led to a marked suppression of activity (Figure 2A).

**Figure 3:**
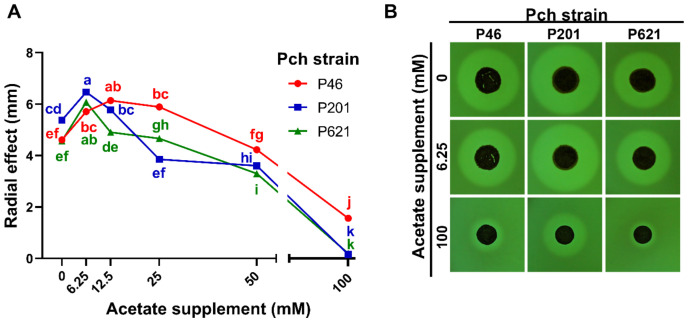
Cellulase enzymatic activity of P46, P201 and P621 Pch strain colonies as a function of acetate supplement concentration in the 0–100 mM range. (**A**) Cellulase activity radius of Pch colonies plotted against acetate supplement concentration. Letters mark significantly (*p* < 0.05) differing datasets according to ANOVA. Different colours correspond to the different fungal strains. (**B**) Representative photographs of Pch colonies grown on YS2 amended with 0.1% *w*/*v* carboxymethyl cellulose at 25 °C for 7 days. Colonies were stained with Gram’s iodine.

Cellulase activity followed the same trend as amylase, showing maximal activity at low (6.25–12.5 mM) acetate concentrations and inhibition at ≥25 mM levels (Figure 3A), with clearly detectable activity zones around the colonies (Figure 3B).

**Figure 4:**
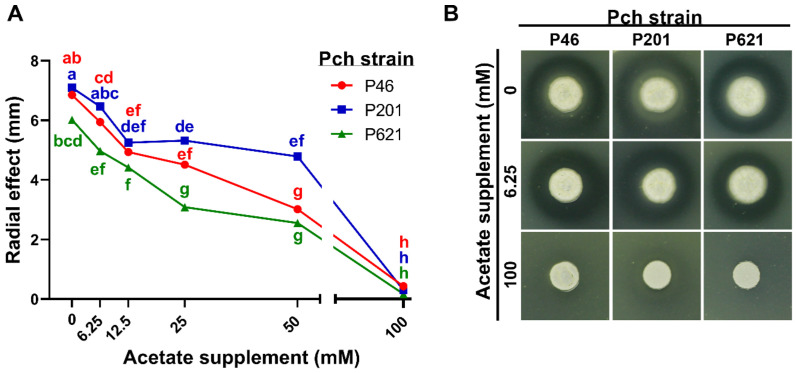
Protease enzymatic activity of P46, P201 and P621 Pch strain colonies as a function of acetate supplement concentration in the 0–100 mM range. (**A**) Protease activity radius of Pch colonies plotted against acetate supplement concentration. Letters mark significantly (*p* < 0.05) differing datasets according to ANOVA. Different colours correspond to the different fungal strains. (**B**) Representative photographs of Pch colonies grown on YS2 amended with 10% *v*/*v* skim milk at 25 °C for 7 days.

Protease activity zones of Pch strains’ colonies (Figure 4B) showed a clear negative correlation with the amount of supplemented acetate at all examined concentrations (Figure 4A).

**Figure 5:**
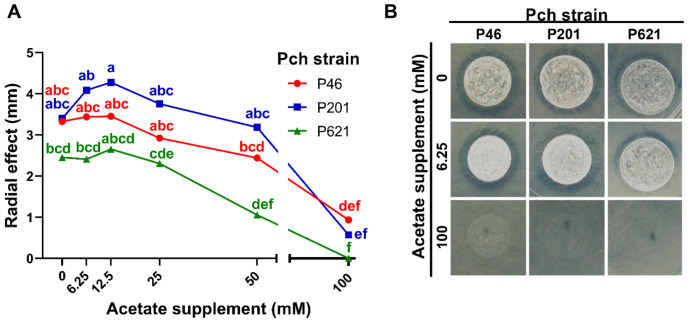
Esterase enzymatic activity of P46, P201 and P621 Pch strain colonies as a function of acetate supplement concentration in the 0–100 mM range. (**A**) Esterase activity radius of Pch colonies plotted against acetate supplement concentration. Letters mark significantly (*p* < 0.05) differing datasets according to ANOVA. Different colours correspond to the different fungal strains. (**B**) Representative photographs of Pch colonies grown on YS2 amended with 0.1% *v*/*v* tributyrin at 25 °C for 7 days.

Esterase activity exhibited moderate, insignificant enhancement at low (6.25–12.5 mM) acetate levels but decreased at >25 mM concentrations (Figure 5A), with barely detectable activity around Pch colonies at 100 mM acetate supplementation (Figure 5B).

**Figure 6:**
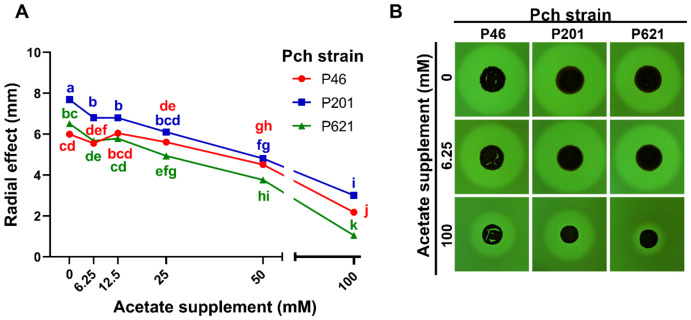
Pectinase enzymatic activity of P46, P201 and P621 Pch strain colonies as a function of acetate supplement concentration in the 0–100 mM range. (**A**) Pectinase activity radius of Pch colonies plotted against acetate supplement concentration. Letters mark significantly (*p* < 0.05) differing datasets according to ANOVA. Different colours correspond to the different fungal strains. (**B**) Representative photographs of Pch colonies grown on YS2 amended with 0.1% *v*/*v* pectin at 25 °C for 7 days. Colonies were stained with Gram’s iodine.

Pectinase activity consistently declined with increasing acetate supplement concentrations (Figure 6A), though the activity can be clearly detected in the case of all Pch strains and all applied acetate supplement concentrations (Figure 6B).

### 3.3. Effect of Acetate on Phenolic Compound-Degrading Ability of Pch

**Figure 7:**
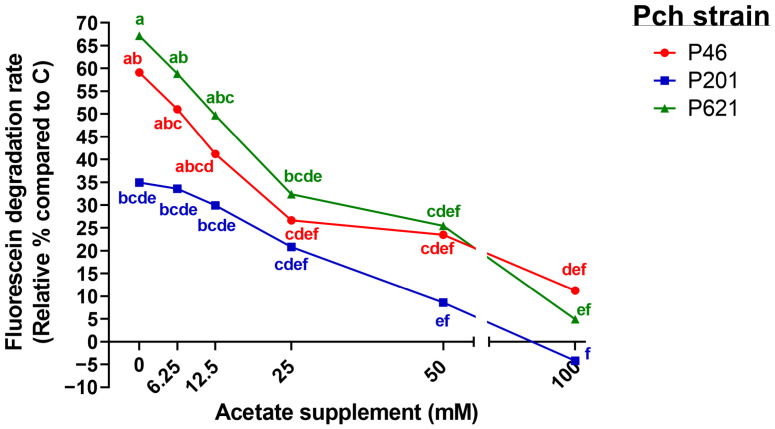
Phenolic compound-degrading ability of P46, P201 and P621 Pch strain colonies as a function of acetate supplement concentration in the 0–100 mM range. Letters mark significantly (*p* < 0.05) differing datasets according to ANOVA. Different colours correspond to the different fungal strains. The control group (C) consisted of a fluorescein-containing medium without Pch, in which the highest fluorescence signal was detected. The figure shows the extent of fluorescein degradation expressed as a percentage relative to C.

The phenolic-degrading capacity of all three examined Pch strains showed a decreasing value with an increasing concentration of acetate supplementation (Figure 7). Notably, a significant deviation from the linear dose–response curve can be observed at 25 mM acetate supplementation in the case of P46 and P621 strains.

### 3.4. Influence of Acetate on the Antibacterial Activity of Pch

**Figure 8:**
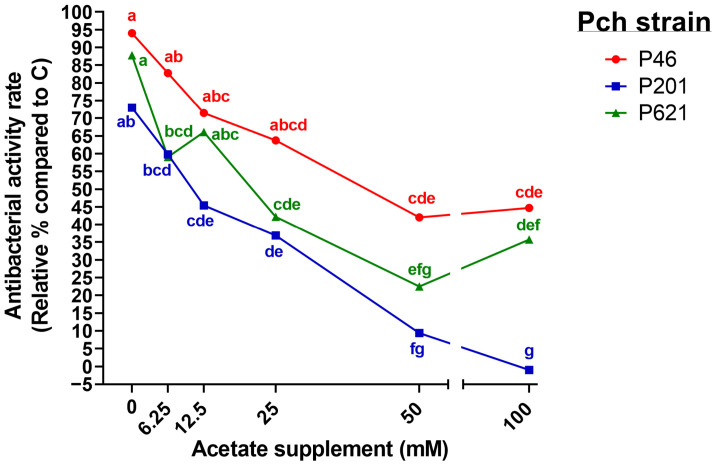
Antibacterial activity of P46, P201 and P621 Pch strain colonies as a function of acetate supplement concentration in the 0–100 mM range. Letters mark significantly (*p* < 0.05) differing datasets according to ANOVA. Different colours correspond to the different fungal strains. The control group consisted of a Pch-free sample containing bacteria, in which the highest optical density was detected. The figure shows the extent of antibacterial activity expressed as a percentage (higher percentages indicate lower bacterial abundance).

Across the tested acetate series, antibacterial activity against a *Pseudomonas* sp. isolate decreased progressively as acetate concentration increased in all three tested Pch strains (Figure 8).

## 4. Discussion

**Figure 9:**
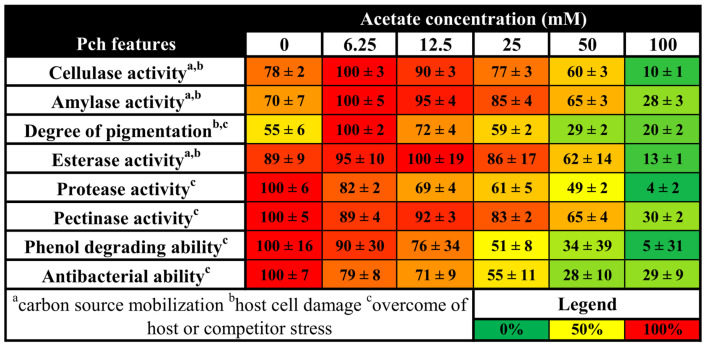
Heat map visualization of the average percent expression of Pch virulence traits in the presence of 0–100 mM acetate supplementation. Values represent the mean percental activities measured for the three Pch strains, along with their standard deviations, considering the highest mean value in each row as 100%. Letters mark suggested biological functions of the different traits.

The results of this study are consistent with a role for acetate beyond that of a metabolic byproduct, potentially acting as a signal that influences the expression of virulence-associated traits in Pch by possibly reflecting the cell density/colonization phase of the fungus. The data reveal diverse dose–response patterns across the examined virulence-associated traits of the fungus, which are summarized in Figure 9. To facilitate biological interpretation, the assessed traits are grouped according to their putative functions: (a) *carbon source mobilization* from host tissues, (b) *host cell damage*, and (c) *overcoming host or competitor stress* (Figure 9). The acetate-response profiles of these traits resolve into three patterns: Gaussian responses (cellulase and amylase activities, pigmentation), an initially flat profile followed by progressive decline (esterase activity), and a monotonic decrease across the entire acetate supplement range (protease and pectinase activities, phenolic compound degradation, antibacterial activity).

Amylase and cellulase activities both peaked at low acetate concentrations (6.25–12.5 mM) and declined at higher levels. These hydrolases contribute primarily to *carbon source mobilization*, releasing soluble sugars from the starch reserves of xylem parenchyma and from the cellulosic component of plant cell walls. The biological relevance of these substrates for Pch is supported by the marked starch depletion observed in Pch-infected wood [5] and the ability of the fungus to degrade crystalline cellulose [37]. Concurrently, cellulolytic and amylolytic activity contributes to *host cell damage* through cell-wall breach and tissue maceration, enabling hyphal expansion. Comparative genomic analyses indicate that Pch retains gene families encoding such carbohydrate-active enzymes [31], and transcriptomic profiling of Pch-*V. vinifera* callus co-cultures have documented the *in planta* induction of glycoside hydrolases [32]. The proposed QS-mediated upregulation of these enzymes in Pch mirrors findings in *C. albicans*, where the QS molecule farnesol induced phospholipase activity, probably to damage host cells and obtain a carbon source [43]. This “low cell density/high activity” state may tentatively be interpreted as reflecting an early colonization phase, during which expansive growth, biomass accumulation, and niche establishment would be favoured. These processes are expected to depend on efficient carbon mobilization and assimilation from the host. This framework remains a working hypothesis that has not been directly tested with infection time-course data.

Pigmentation followed a Gaussian response comparable to that of polysaccharide-degrading enzymes. Fungal melanisation is a well-documented protective trait against UV radiation, reactive oxygen species generated by host immunity, and lytic enzymes [27,28], placing it within the *overcoming host or competitor stress* functional category. In Pch, however, the interpretation is more nuanced: scytalone and isosclerone—two well-characterized intermediates of the DHN-melanin biosynthetic pathway—have been identified as phytotoxic metabolites of this pathogen [29]. Enhanced pigmentation therefore implies elevated flux through a pathway whose intermediates compromise host cell viability, indirectly facilitating carbon-source utilization through *host cell damage*. Notably, all three traits with Gaussian response to acetate supplementation (amylase, cellulase, pigmentation) returned to their acetate-free baseline values at about 25 mM acetate concentration. Given that Karácsony et al. [12] reported a maximal acetate accumulation of approximately 13 mM within Pch biofilms, the 6.25–25 mM range applied here lies within physiologically relevant signalling concentrations rather than reflecting an unnatural stress regime. We therefore suggest that the acetate responses of Pch in this range are more compatible with a putative QS-mediated regulatory function than with a generalized stress response, although direct mechanistic confirmation (e.g., via QS-pathway interference or receptor identification) is still lacking. Above 25 mM acetate, we instead suggest a generalized acetic-acid stress response interpretation [44,45], where progressive activity loss is consistent with the cytotoxic effects of acetate on eukaryotic cells. Therefore, at higher concentrations (≥50 mM), the growth inhibitory activity of acetate is more likely to be responsible for the lower enzyme activities and pigmentation rather than the signal function of the molecule. In agreement with this hypothesis, a significant decrease in all measured parameters was observed in the presence of 50 and 100 mM acetate (Figure 9). However, to clearly see what concentrations of acetate are relevant in Pch physiology and pathogenesis, further studies are needed to measure this parameter in Pch-infected grapevine.

Esterase activity represents the first qualitative deviation from the Gaussian acetate-response profile: it was not significantly affected at low acetate concentrations but declined progressively above 25 mM. The concentration of lipids in angiosperm woody tissues falls in the sub-nanomolar range [46], rendering them insufficient carbon sources for endophytic microbes. In addition, the abundance of polysaccharides in xylem tissue makes carbohydrate-active enzymes a more energetically favourable route for nutrient acquisition during wood colonization. We therefore interpret extracellular esterase activity in Pch as primarily contributing to *host cell damage*—for instance through degradation of plant membrane lipids—rather than to carbon mobilization. The transcriptomic detection of an extracellular lipase upregulated during Pch-*V. vinifera* callus interaction is consistent with such a role during host engagement [32].

Protease activity declined monotonically with increasing acetate supplementation. Upon fungal invasion, grapevine tissues accumulate a battery of pathogenesis-related (PR) proteins—including chitinases, β-1,3-glucanases, and thaumatin-like proteins—exhibiting direct antifungal activity [47,48]. Secreted fungal proteases capable of degrading or inactivating these defence proteins constitute a recognized fungal counter-defence strategy [49]. The maintenance of high protease output at low fungal densities—when host PR responses are most acutely deployed in the early infection court—and its progressive downregulation as cell density (and acetate concentration) increases is consistent with such a defensive role rather than with a nutritional one, suggesting the functional classification of protease activity as *overcoming host stress*. Similar downregulation of aspartyl proteases by the QS-molecule farnesol was observed in *C. albicans* [50], supporting a broader trend of cell density-dependent control of protease secretion in pathogenic fungi.

Pectinase activity also decreased monotonically with supplemented acetate concentration. Although pectinases are conventionally classified among the cell wall-degrading enzymes that contribute to *carbon source mobilization* and *host cell damage*, mature xylem contains relatively little pectin compared to cellulose and lignin. While there is no data on grapevine wood pectin content, it is only 1–4% *m*/*v* in eucalyptus [51]. In grapevine, pectin-rich gels and the walls of newly formed tyloses are actively deposited within xylem vessels as part of the host’s vascular defence response [5]. Pouzoulet et al. [5] explicitly attribute Pch’s ability to progress within occluded vessels to its capacity to colonize pectin-rich gel pockets and outer tylose walls. Pch pectinases therefore likely act principally to dismantle these inducible host barriers—an *overcoming host stress* function—rather than to release significant carbohydrate substrates. Sustained pectinolytic activity at low fungal cell densities (signalled by low extracellular acetate), when host occlusion responses are most active, and its downregulation at higher acetate concentrations are compatible with—though do not directly establish—a stage-dependent regulatory model, which would require *in planta* validation.

The phenolic compound-degrading capacity of Pch declined progressively with acetate concentration. Grapevine xylem accumulates stilbenes—most notably trans-resveratrol and its viniferin oligomers—together with flavonoids and hydroxycinnamic acids that function as antifungal phytoalexins in response to pathogen invasion [35,36]. In Botryosphaeriaceae GTD pathogens, stilbene metabolization is mediated by extracellular oxidoreductases, including laccases and peroxidases [52]. However, Pch secretes lignin-modifying oxidoreductases at notably lower levels than other GTD-associated fungi [33]. A non-enzymatic alternative is more compatible with our data: Osti et al. [37] demonstrated that Pch generates hydroxyl radicals via secreted low-molecular-weight phenolic metabolites in a Fenton-type chemistry reminiscent of brown-rot wood decay. Such oxidative chemistry can non-specifically degrade host phenolic defences, providing Pch with a means to neutralize the chemical arm of host resistance, contributing to *overcoming-host-stress* function. The downregulation of this capacity at high acetate concentrations would be consistent with a reallocation of resources once the chemical microenvironment has been neutralized at the abundant niche-colonization phase, although this remains to be confirmed *in planta*.

The antibacterial activity of Pch against a *Pseudomonas* sp. isolate was strongest in the absence of supplemental acetate and decreased monotonically as acetate concentration increased. To our knowledge, this is the first experimental demonstration of antibacterial activity attributable to Pch metabolites. Grapevine xylem harbours complex bacterial communities, and competitive interactions among co-inhabiting microorganisms likely influence colonization dynamics and plant health [18]. Notably, several *Pseudomonas* sp. isolates were proven to be efficient antagonists of Pch [53]. The polyketide-derived and naphthalenone secondary metabolites previously characterized in Pch [29] represent plausible candidates for the observed antibacterial activity, although the specific compound(s) remain to be identified. As with phenolic compound degradation, the suppression of this competitor-targeting function at elevated acetate concentrations would be consistent with a reallocation of metabolic effort once competition has subsided, an *overcoming-competitor-stress* trait that we tentatively associate with an early establishment phase, pending further validation.

## 5. Conclusions

Collectively, these findings are consistent with a model in which Pch may sense its own population state through acetate levels, although the underlying sensing mechanism has not been identified here. The number of studies on analogous processes in other fungal species is limited. In fact, acetate is referred to as a quorum sensing molecule in a single study based on the examination of 2,3-butanediol production in *Saccharomyces cerevisiae* [54]. An additional study suggests that the acetate signal promotes appressoria formation in *Magnaporthe oryzae*. However, this latter phenomenon is not related to cell population density, since it is based on the acetate released by a chitin deacetylase at the hyphal tips [55]. These latter papers have no data on the possible signal transduction mechanism involved in acetate sensing. Meanwhile, these studies related to fungal species taxonomically distant from Pch suggest that acetate-mediated autoregulation may play an important yet hidden role in several fungi.

Based on our in vitro results, we propose a hypothetical model in which Pch may alternate between two functional states, regulated by the sensing of acetate produced by the fungus. The early colonization state is characterized by high carbohydrate-degrading enzymatic activity, intense bacterial antagonism, and active phenolic detoxification. The higher expression of these traits is expected to facilitate the breaching of host defences, exclusion of competitors, and acquisition of carbon for biomass production. The persistent state is characterized by reduced extracellular enzyme secretion and metabolic conservation, alongside the previously characterized biofilm formation. This latter growth strategy supports the long-term persistence of Pch in an occupied niche. This proposed model has not been directly tested here and would require *in planta* time-course or transcriptomic validation. In addition, further experiments aiming to uncover molecules contributing to acetate signalling in Pch may further extend our understanding of Pch pathogenesis and may help to identify similar processes in other fungi. If confirmed, such regulatory flexibility could help balance the energetic cost of virulence factor expression against the need for long-term persistence in the grapevine trunk, potentially contributing to the chronic course of esca disease; this remains a hypothesis for future investigation.

## Figures and Tables

**Table 1:** Data of fungal and bacterial strains used in this study.

Strain ID	Species	Origin	Isolation Place	Isolation Year	Reference	ITS GenBank Accession
P46	*Phaeomoniella chlamydospora*	Grapevine wood	Eger, Hungary	2017	[38]	PP510215
P201	*Phaeomoniella chlamydospora*	Grapevine wood	Diósviszló, Hungary	2015	[12]	PP510216
P621	*Phaeomoniella chlamydospora*	Grapevine wood	Egerszólát, Hungary	2015	[12]	PP510217
*Pseudomonas* sp.	*Pseudomonas* sp.	Grapevine wood	Eger, Hungary	2023	This study	PZ256757

## Data Availability

Nucleic acid sequences were uploaded to GenBank. Any other research data are presented in the study or as a Supplementary File.

## Supplementary Materials

The following supporting information can be downloaded at: https://www.mdpi.com/article/10.3390/jof12070539/s1. The raw data of all measurements were uploaded as a Supplementary Table. Table S1: Raw replicate data for pigmentation, cellulase, amylase, pectinase, protease, esterase activities, fluorescein degradation, and antibacterial activity of *Phaeomoniella chlamydospora* strains P46, P201, and P621 under acetate supplementation (0–100 mM).
